# Drug prescribing during the last year of life in very old people with diabetes

**DOI:** 10.1093/ageing/afw174

**Published:** 2016-10-15

**Authors:** Shota Hamada, Martin C. Gulliford

**Affiliations:** 1 Department of Primary Care and Public Health Sciences, King's College London, London, UK; 2 National Institute for Health Research (NIHR) Biomedical Research Centre at Guy's and St Thomas’ NHS Foundation Trust, London, UK

**Keywords:** aged, 80 and over, end-of-life care, older people, polypharmacy, type 2 diabetes mellitus

## Abstract

**Objective:**

to evaluate primary care drug utilisation during the last year of life, focusing on antidiabetic and cardiovascular drugs, in patients of advanced age with diabetes.

**Design:**

population-based cohort study.

**Setting:**

primary care database in the UK.

**Subjects:**

patients with type 2 diabetes who died at over 80 years of age between 2011 and 13.

**Methods:**

main outcome measures included proportions of patients prescribed different classes of drugs, comparing the first (Q1) and the fourth quarters (Q4) of the last year of life.

**Results:**

the study included 5,324 patients, with the median age 86 years and 50% female. Three-fourths of the patients received five or more drugs, and the total number of drugs prescribed was almost stable at 6.2 ± 3.1 (mean ± SD) during the last year of life. Substantial proportions of patients were treated with antidiabetic drugs (78%), antihypertensive drugs (76%), statins (62%) and low-dose aspirin (46%) in Q1. Prescribing of these drugs slightly decreased by 3–8% in Q4. There were increases in prescribing of anti-infectives (35% in Q1 to 50% in Q4), drugs for nervous system (63% to 73%), drugs for respiratory system (24% to 33%) and systemic hormonal drugs (22% to 27%).

**Conclusion:**

patients of advanced age with type 2 diabetes were often treated with antidiabetic and cardiovascular drugs even when approaching death. More research is needed to generate evidence to guide optimal drug utilisation for older people with a limited life expectancy.

## Introduction

The number of very old people is increasing, and both the prevalence of chronic diseases, including diabetes, and the intensity of drug treatment have been increasing in this population [1]. Patients with diabetes are commonly treated with multiple classes of drugs to control risk factor values and to reduce cardiovascular risk, consistent with guideline recommendations [2]. These recommendations are often applied in very old people over 80 years of age [3]. Drugs to lower cardiovascular risk are often prescribed for several decades or even life-long, despite lack of evidence for such a long-term therapy [4]. Some observational studies suggest that low levels of cardiovascular risk factors, including HbA1c, blood pressure and cholesterol, may sometimes be associated with higher mortality in very old patients with type 2 diabetes [5]. As patients approach the end-of-life, care to control symptoms and to improve well-being become important. At present, there is insufficient evidence to guide end-of-life care for patients with diabetes [6, 7]. This study aimed to evaluate primary care drug utilisation during the last year of life, focusing on antidiabetic and cardiovascular drugs, in patients of advanced age with diabetes.

## Methods

### Patients

Patients 80 years or older with type 2 diabetes who died between 2011 and 13 were sampled from the UK Clinical Practice Research Datalink (CPRD). CPRD collects electronic health records from primary care across the UK, and participants are representative of general population [8]. Patients with type 2 diabetes were selected based on diagnoses of diabetes, HbA1c values and prescriptions of antidiabetic drugs. Patients who consulted their general practitioners at least once every 3 months in the last year of life were selected to include community-dwelling people who were generally managed in primary care. Patients who ended their registration with a CPRD general practice before death were excluded. The study was approved by the CPRD Independent Scientific Advisory Committee (ISAC Protocol 14_053).

### Measurements

All drugs prescribed, except for topical drugs for local effects, vitamins/minerals, nutritional products, herbal remedy or vaccines, were evaluated regardless of duration of treatment. We focused on antidiabetic drugs and cardiovascular drugs for prevention and treatment of cardiovascular diseases (CVD), including antihypertensive drugs, statins and low-dose aspirin. Numbers of drug classes for individual patients were evaluated according to the third level (pharmacological subgroup; e.g. A10A insulins and analogues) of the WHO ATC classification.

### Analysis

Baseline characteristics were evaluated at the start of observation (i.e. 365 days before death dates). Frequencies and proportions of patients prescribed each class of drugs were calculated in the first (Q1; 12–9 months before death) and the fourth quarters (Q4; 3 months before death to death dates) of the last year of life. Analysis was performed in overall and according to a history of CVD (coronary heart disease and stroke). Changes in prescriptions from Q1 to Q4 and differences in prescriptions between patients with CVD and those without were tested. Total numbers of drug classes were counted on the monthly basis.

## Results

### Characteristics of patients

The study included 5,324 patients, with the median age 86 years and 50% female (Table 1). The median duration of diabetes was 10 years. Half of the patients had a history of CVD. Two-thirds of the patients had estimated glomerular filtration rate (eGFR) of <60 mL/min/1.73 m^2^, calculated using the Chronic Kidney Disease Epidemiology Collaboration equation [9]. A majority of the patients were managed their cardiovascular risk factors below relaxed targets for HbA1c (<8.5% or <69 mmol/mol), blood pressure (systolic <150 and diastolic <90 mmHg) and total cholesterol (<5.0 mmol/L).

**Table 1.** Characteristics of patients (*N* = 5,324)

Characteristics		*n* (%)/Median (IQR)
Sex	Male	2,656 (50)
	Female	2,668 (50)
Age (years)		86 (83–89)
Duration of diabetes (years)		10 (6–16)
eGFR (mL/min/1.73 m^2^)	<30	773 (15)
	30–44	1,290 (24)
	45–59	1,306 (25)
	≥60	1,955 (37)
HbA1c (%, mmol/mol)	<6.5 (<48)	1,164 (22)
	6.5–7.4 (48–57)	1,901 (36)
	7.5–8.4 (58–68)	876 (16)
	≥8.5 (≥69)	1,383 (26)
Systolic/diastolic blood pressure (mmHg)^a^	<130 and <70	1,156 (23)
	<140 and <80 (but ≥130 or ≥70)	1,770 (36)
	<150 and <90 (but ≥140 or ≥80)	1,236 (25)
	≥150/90	817 (16)
Total cholesterol (mmol/L)	<3.0	531 (10)
	3.0–3.9	1,721 (32)
	4.0–4.9	1,431 (27)
	≥5.0	1,641 (31)
Comorbidities	Cardiovascular disease	2,710 (51)
	Coronary heart disease	2,260 (42)
	Stroke	815 (15)
	Cancer	1,875 (35)
	Dementia/Cognitive decline	673 (13)

eGFR, estimated glomerular filtration rate; IQR, interquartile range.

^a^Excluding 345 patients with missing values for blood pressure.

### Antidiabetic and cardiovascular drugs

Most of the patients (78%) were treated with antidiabetic drugs during the last year of life (Table 2). Metformin and sulphonylureas were prescribed frequently at similar levels in overall. Metformin was less frequently prescribed in patients with decreased renal function: among patients on antidiabetic medications, although 70% of the patients with eGFR ≥60 mL/min/1.73 m^2^ received metformin, 35% or 14% of the patients with eGFR <45 or <30 mL/min/1.73 m^2^, respectively, received this drug. Insulins were more frequently prescribed in patients with a history of CVD compared with those without (*P* < 0.001). A slight decrease (6%) in prescribing of antidiabetic drugs were observed from Q1 to Q4 (*P* < 0.001). Substantial proportions of patients were treated with antihypertensive drugs (76%), statins (62%) and low-dose aspirin (46%) in Q1. Patients with a history of CVD had more intensive pharmacological treatment with the cardiovascular drugs than those without (all *P* < 0.001). Prescribing of the cardiovascular drugs decreased by 3–8% from Q1 to Q4 as patients approached death (all *P* < 0.001).

**Table 2.** Prescriptions of antidiabetic and cardiovascular drugs in the last year of life

	Overall (*N* = 5,324)			No history of CVD (*N* = 2,614)			History of CVD (*N* = 2,710)		
	Q1	Q4	*P* value	Q1	Q4	*P* value	Q1	Q4	*P* value
***Antidiabetic drugs***	***4,164 (78)***	***3,816 (72)***	***<0.001***	***2,048 (78)***	***1,864 (71)***	***<0.001***	***2,116 (78)***	***1,952 (72)***	***<0.001***
Insulins	791 (15)	768 (14)	0.189	321 (12)	310 (12)	0.351	470 (17)	458 (17)	0.355
Metformin	2,316 (44)	2,054 (39)	<0.001	1,186 (45)	1,046 (40)	<0.001	1,130 (42)	1,008 (37)	<0.001
Sulphonylureas	2,215 (42)	1,937 (36)	<0.001	1,130 (43)	989 (38)	<0.001	1,085 (40)	948 (35)	<0.001
Other antidiabetic drugs	433 (8)	388 (7)	0.002	242 (9)	210 (8)	0.002	191 (7)	178 (7)	0.173
***Antihypertensive drugs***	***4,053 (76)***	***3,629 (68)***	***<0.001***	***1,882 (72)***	***1,647 (63)***	***<0.001***	***2,171 (80)***	***1,982 (73)***	***<0.001***
RAS blockers	3,037 (57)	2,510 (47)	<0.001	1,387 (53)	1,133 (43)	<0.001	1,650 (61)	1,377 (51)	<0.001
β-blockers	1,648 (31)	1,676 (31)	0.205	552 (21)	559 (21)	0.622	1,096 (40)	1,117 (41)	0.215
Ca-channel blockers	1,554 (29)	1,304 (24)	<0.001	797 (30)	676 (26)	<0.001	757 (28)	628 (23)	<0.001
Thiazide diuretics	700 (13)	498 (9)	<0.001	428 (16)	292 (11)	<0.001	272 (10)	206 (8)	<0.001
***Statins***	***3,290 (62)***	***2,850 (54)***	***<0.001***	***1,389 (53)***	***1,179 (45)***	***<0.001***	***1,901 (70)***	***1,671 (62)***	***<0.001***
***Low-dose aspirin***	***2,461 (46)***	***2,281 (43)***	***<0.001***	***1,006 (39)***	***943 (36)***	***0.001***	***1,455 (54)***	***1,338 (49)***	***<0.001***

CVD, cardiovascular diseases. Data are shown as frequencies (%).

Q1, 12–9 months before death; Q4, 3 months before death to death dates.

RAS blockers: drugs affecting the renin–angiotensin system.

### Overall prescriptions

Drugs in ATC main groups B (e.g. low-dose aspirin and warfarin) and C (e.g. antihypertensive drugs, statins, diuretics and drugs for angina) were less prescribed in Q4 compared with Q1 (B, 66% in Q1 to 61% in Q4; C, 90% to 84%). Drugs in group J (anti-infectives for systemic use) were increased most (35% in Q1 to 50% in Q4), followed by group N (nervous system, 63% to 73%), group R (respiratory system, 24% to 33%) and group H (systemic hormonal preparations; e.g. thyroid hormones and corticosteroids, 22% to 27%). Other frequently prescribed groups of drugs (group A, 91% to 90%; G, 15% to 16% and M, 22% to 21%) were prescribed at similar levels between Q1 and Q4.

In patients who received any of the drugs of interest in a given month (79–92% of the overall patients), the total number of drugs prescribed was almost stable at 6.2 ± 3.1 (mean ± SD) during the last year of life, and 55% or 18% of the patients received 5–9 or 10 different classes of drugs in a month.

## Discussion

This study showed that very old people with diabetes received intense pharmacological treatment with antidiabetic and cardiovascular drugs during the last year of life. We caution that the study cohort were selected because they died, but death may not always have been clearly anticipated. Their care may not have been recognised as being for the end-of-life. Prescribing might be justified if patients were expected to live longer than the time needed to obtain benefits from the drugs. Even if antidiabetic medications do not exert preventive effects on long-term complications due to a short period of follow-up, blood glucose lowering may reduce risks for infections and dehydration at the end-of-life phase. However, avoidance of diabetes-related emergent complications, such as hypoglycaemia, is an important aspect of care for older patients with diabetes [7]. Potential overtreatment of blood glucose and blood pressure in older people were recently reported in the USA [10, 11]. However, clinicians may be reluctant to de-escalate treatment in patients with evidence of complications. These findings may be partly supported by the perception of a difficulty in making decisions on deintensification of drug treatment for future risk reduction [12]. Polypharmacy was commonly observed in this study, which could be a great concern because of increased risks of drug–drug interactions and adverse events [13, 14]. Further research on reducing inappropriate drugs in patients approaching the end-of-life is needed [15].

‘Deprescribing’ is a medication review process of discontinuing drugs to improve outcomes and decrease risks associated with polypharmacy [16]. So far, some recommendations on end-of-life care in patients with diabetes have been provided based on available clinical evidence [6, 7, 17]. Evidence supporting decision-making of discontinuation of drugs may be generated from clinical studies or observational studies with careful consideration of confounding by indication. A recent randomised controlled trial suggested that discontinuation of statins appeared to be safe, might improve quality of life and reduced medication costs in patients with a limited life expectancy [18]. However, some issues were indicated in the study [19, 20], which can be also challenges for deprescribing trials in the future. Further research is required on how to generate and utilise evidence to reduce the inappropriate use of drugs in such populations.

There were some limitations in this study although the CPRD provided a representative sample of the UK general population. First, we did not separate patients based on an estimated life expectancy or analyse causes of deaths, which made it difficult to evaluate appropriateness of prescribing. Second, we could not know if patients actually took their medications prescribed from the database. Some patients might have discontinued taking the drugs prescribed, especially in the last few months of life. Third, we analysed data from primary care, including some over-the-counter drugs recorded in the CPRD but did not include drugs prescribed in hospitals or care facilities. Finally, the study cohort managed in primary care might have different health status and intensity of pharmacological treatment compared with those managed in other settings, which may limit generalisability of the results.

## Conclusion

Patients with type 2 diabetes were often treated with antidiabetic and cardiovascular drugs even in their advanced ages approaching death. Deprescribing might be considered for this population when patients are not expected to have enough time to obtain benefits from the medications or patients are at substantial risks of symptomatic adverse events. More research is needed to generate evidence to guide optimal drug utilisation for older people with a limited life expectancy.

**Key points**

- Prescribing of medications during the last year of life in primary care was evaluated in older patients with diabetes.

- Patients with diabetes were often treated with antidiabetic and cardiovascular drugs at the end-of-life.
- Discontinuation of some of drugs might be considered to improve outcomes and decrease risks of adverse events.
- More research is needed to generate evidence to guide optimal drug utilisation for this vulnerable population.
